# 2,4-Dichloro­phenyl 4-bromo­benzene­sulfonate

**DOI:** 10.1107/S1600536809042950

**Published:** 2009-10-23

**Authors:** Nagarajan Vembu, Frank R. Fronczek

**Affiliations:** a Department of Chemistry, Urumu Dhanalakshmi College, Tiruchirappalli 620 019, India; b Department of Chemistry, Louisiana State University, Baton Rouge, LA 70803-1804, USA

## Abstract

In the title mol­ecule, C_12_H_7_BrCl_2_O_3_S, the dihedral angle between the two benzene rings is 55.18 (5)°. The notable inter­molecular contacts include C—H⋯O and π–π inter­actions [centroid–centroid distances = 4.037 (1) and 3.349 (1) Å].

## Related literature

For a detailed account of the mol­ecular and supra­molecular architectures of aromatic sulfonates, see Vembu *et al.* (2007 ▶). For a general background to aromatic sulfonates, see: Yachi *et al.* (1989 ▶): Spungin *et al.* (1992 ▶); Tharakan *et al.* (1992 ▶); Alford *et al.* (1991 ▶); Jiang *et al.* (1990 ▶); Narayanan & Krakow (1983 ▶). For the criteria to describe C—H⋯O inter­actions, see: Desiraju & Steiner, (1999 ▶) and for the classification of aromatic stacking inter­actions, see: Spek (2009 ▶).
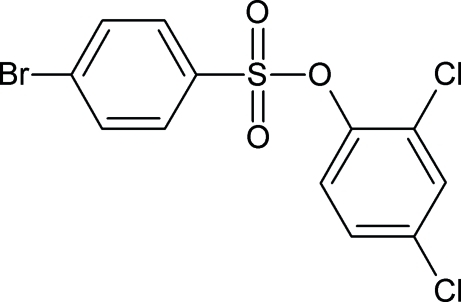


## Experimental

### 

#### Crystal data

C_12_H_7_BrCl_2_O_3_S
*M* *_r_* = 382.05
Triclinic, 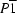

*a* = 7.2955 (10) Å
*b* = 8.3955 (11) Å
*c* = 11.1251 (15) Å
α = 95.737 (8)°
β = 98.645 (7)°
γ = 96.231 (8)°
*V* = 664.98 (15) Å^3^
*Z* = 2
Mo *K*α radiation
μ = 3.65 mm^−1^
*T* = 90 K
0.17 × 0.10 × 0.07 mm

#### Data collection

Nonius KappaCCD diffractometer with Oxford Cryostream
Absorption correction: multi-scan (*HKL* *SCALEPACK*; Otwinowski & Minor 1997 ▶) *T*_min_ = 0.576, *T*_max_ = 0.784
18308 measured reflections
4745 independent reflections
4091 reflections with *I* > 2σ(*I*)
*R*_int_ = 0.025

#### Refinement

*R*[*F* ^2^ > 2σ(*F* ^2^)] = 0.031
*wR*(*F* ^2^) = 0.074
*S* = 1.05
4745 reflections
200 parameters
All H-atom parameters refined
Δρ_max_ = 0.42 e Å^−3^
Δρ_min_ = −0.76 e Å^−3^

### 

Data collection: *COLLECT* (Nonius, 2000 ▶); cell refinement: *DENZO* and *SCALEPACK* (Otwinowski & Minor, 1997 ▶); data reduction: *DENZO* and *SCALEPACK*; program(s) used to solve structure: *SHELXS97* (Sheldrick, 2008 ▶); program(s) used to refine structure: *SHELXL97* (Sheldrick, 2008 ▶); molecular graphics: *PLATON* (Spek, 2009 ▶); software used to prepare material for publication: *SHELXL97*.

## Supplementary Material

Crystal structure: contains datablocks I, global. DOI: 10.1107/S1600536809042950/om2286sup1.cif

Structure factors: contains datablocks I. DOI: 10.1107/S1600536809042950/om2286Isup2.hkl

Additional supplementary materials:  crystallographic information; 3D view; checkCIF report

## Figures and Tables

**Table 1:** Hydrogen-bond geometry (Å, °)

*D*—H⋯*A*	*D*—H	H⋯*A*	*D*⋯*A*	*D*—H⋯*A*
C2—H2⋯O3^i^	0.95 (3)	2.58 (3)	3.482 (2)	160 (2)
C11—H11⋯O1^ii^	0.91 (2)	2.52 (2)	3.390 (2)	160 (2)

Symmetry codes: (i) 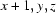; (ii) 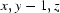.

**Fig. 1.**
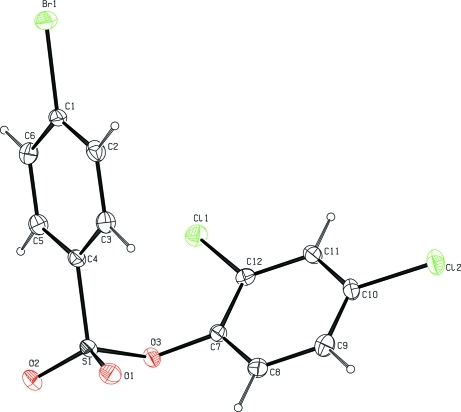
The asymmetric unit with the atoms labelled and displacement ellipsoids depicted at the 50% probability level for all non-H atoms. H-atoms are drawn as spheres of arbitrary radius.

## supplementary crystallographic information

### Comment

Aromatic sulfonates are used in monitoring the merging of lipids (Yachi *et al.*, 1989) and in many other fields (Spungin *et al.*, 1992; Tharakan *et al.*,1992; Alford *et al.*, 1991; Jiang *et al.*, 1990; Narayanan & Krakow, 1983). An X-ray study of the title compound was undertaken in order to determine its crystal and molecular structure owing to the biological importance of its analogues. The molecular structure is shown in Fig. 1.

The C4–S–O3–C7 torsion angle of 65.86 (14)° corresponds to +synclinal conformation; as expected the dihedral angle between the mean planes of the 2,4-dichlorophenyl and bromobenzene rings of 55.18 (5)° shows that the two rings are not coplanar. This is similar to the situation reported by us for other aromatic sulfonates (Vembu *et al.* 2007 and references cited therein).

The crystal structure exhibits weak intermolecular C—H···O interactions (Desiraju & Steiner, 1999) (Table 1). There are two face to face π···π aromatic stacking interactions. The coordinates of *Cg*1···*Cg*1 (-*x*, 1 - *y*, 1 - *z*) at 4.037Å are α = 0.00, β = 24.13, γ = 24.13, the two perpendicular distances involving the aromatic rings being 3.684 Å. The coordinates of *Cg*2···*Cg*2 (-1 - *x*, -*y*, 2 - *z*) at 3.751Å are α = 0.03, β = 26.79, γ = 26.79, the two perpendicular distances involving the aromatic rings being 3.349 Å. *Cg*1 is the centroid of the aromatic ring formed by the atoms C1, C2, C3, C4, C5 & C6, *Cg*2 is the centroid of the aromatic ring formed by the atoms C7, C8, C9, C10, C11 & C12. α is the dihedral angle between the planes of the two aromatic rings, β is the angle of the aromatic ring formed by the atoms C1—C6 through the aromatic ring formed by the atoms C7—C12, γ is the angle of the vector through the centroids of the planes of the two aromatic rings and normal to the plane of the aromatic ring formed by C7—C12 (Spek, 2009).

### Experimental

4-Bromobenzenesulfonyl chloride (10 mmol), dissolved in acetone (10 ml), was added dropwise to 2,4-dichlorophenol (10 mmol) in aqueous NaOH (8 ml, 5%) with constant stirring. The precipitate (6.5 mmol, yield 65%) was filtered and recrystallized from aqueous ethanol.

### Refinement

All H-atoms were located in difference maps and their positions and isotropic displacement parameters freely refined.

### Crystal data

**Table:**

C_12_H_7_BrCl_2_O_3_S	*Z* = 2
*M**_r_* = 382.05	*F*(000) = 376
Triclinic, *P*1	*D*_x_ = 1.908 Mg m^−^^3^
Hall symbol: -P 1	Melting point: 398 K
*a* = 7.2955 (10) Å	Mo *K*α radiation, λ = 0.71073 Å
*b* = 8.3955 (11) Å	Cell parameters from 4109 reflections
*c* = 11.1251 (15) Å	θ = 2.5–33.6°
α = 95.737 (8)°	µ = 3.65 mm^−^^1^
β = 98.645 (7)°	*T* = 90 K
γ = 96.231 (8)°	Prism, colorless
*V* = 664.98 (15) Å^3^	0.17 × 0.10 × 0.07 mm

### Data collection

**Table:**

Nonius KappaCCD diffractometer with Oxford Cryostream	4745 independent reflections
Radiation source: fine-focus sealed tube	4091 reflections with *I* > 2σ(*I*)
graphite	*R*_int_ = 0.025
ω scans with κ offsets	θ_max_ = 33.5°, θ_min_ = 2.9°
Absorption correction: multi-scan (*HKL**SCALEPACK*; Otwinowski & Minor 1997)	*h* = −10→11
*T*_min_ = 0.576, *T*_max_ = 0.784	*k* = −12→12
18308 measured reflections	*l* = −16→17

### Refinement

**Table:**

Refinement on *F*^2^	Primary atom site location: structure-invariant direct methods
Least-squares matrix: full	Secondary atom site location: difference Fourier map
*R*[*F*^2^ > 2σ(*F*^2^)] = 0.031	Hydrogen site location: difference Fourier map
*wR*(*F*^2^) = 0.074	All H-atom parameters refined
*S* = 1.05	*w* = 1/[σ^2^(*F*_o_^2^) + (0.0284*P*)^2^ + 0.6284*P*] where *P* = (*F*_o_^2^ + 2*F*_c_^2^)/3
4745 reflections	(Δ/σ)_max_ = 0.001
200 parameters	Δρ_max_ = 0.42 e Å^−^^3^
0 restraints	Δρ_min_ = −0.76 e Å^−^^3^

### Special details

**Table:**

Geometry. All su's (except the su in the dihedral angle between two l.s. planes) are estimated using the full covariance matrix. The cell su's are taken into account individually in the estimation of su's in distances, angles and torsion angles; correlations between su's in cell parameters are only used when they are defined by crystal symmetry. An approximate (isotropic) treatment of cell su's is used for estimating su's involving l.s. planes.
Refinement. Refinement of *F*^2^ against ALL reflections. The weighted *R*-factor *wR* and goodness of fit *S* are based on *F*^2^, conventional *R*-factors *R* are based on *F*, with *F* set to zero for negative *F*^2^. The threshold expression of *F*^2^ > σ(*F*^2^) is used only for calculating *R*-factors(gt) *etc*. and is not relevant to the choice of reflections for refinement. *R*-factors based on *F*^2^ are statistically about twice as large as those based on *F*, and *R*- factors based on ALL data will be even larger.

### Fractional atomic coordinates and isotropic or equivalent isotropic displacement parameters (Å^2^)

**Table:**

	*x*	*y*	*z*	*U*_iso_*/*U*_eq_	
Br1	0.64701 (3)	0.17437 (2)	0.428948 (18)	0.01931 (6)	
Cl1	0.02233 (6)	0.03498 (5)	0.69619 (4)	0.01838 (9)	
Cl2	0.36290 (6)	−0.08990 (6)	1.13426 (5)	0.02035 (10)	
S1	0.14495 (6)	0.51012 (5)	0.77797 (4)	0.01350 (8)	
O1	0.26445 (19)	0.59769 (16)	0.88237 (13)	0.0180 (3)	
O2	0.00437 (19)	0.58520 (17)	0.70897 (13)	0.0193 (3)	
O3	0.02261 (17)	0.36107 (15)	0.82191 (12)	0.0136 (2)	
C1	0.4977 (2)	0.2748 (2)	0.53130 (17)	0.0139 (3)	
C2	0.5760 (2)	0.3301 (2)	0.65200 (18)	0.0161 (3)	
C3	0.4664 (3)	0.4019 (2)	0.72856 (17)	0.0154 (3)	
C4	0.2818 (2)	0.4176 (2)	0.68176 (16)	0.0129 (3)	
C5	0.2045 (2)	0.3631 (2)	0.56058 (17)	0.0150 (3)	
C6	0.3137 (3)	0.2911 (2)	0.48443 (17)	0.0159 (3)	
C7	0.1140 (2)	0.2578 (2)	0.89617 (16)	0.0125 (3)	
C8	0.1862 (3)	0.3101 (2)	1.01796 (17)	0.0157 (3)	
C9	0.2658 (3)	0.2032 (2)	1.09162 (17)	0.0169 (3)	
C10	0.2681 (2)	0.0454 (2)	1.04188 (17)	0.0148 (3)	
C11	0.1949 (2)	−0.0090 (2)	0.92034 (17)	0.0145 (3)	
C12	0.1179 (2)	0.0995 (2)	0.84746 (16)	0.0129 (3)	
H2	0.702 (4)	0.319 (3)	0.682 (2)	0.023 (6)*	
H3	0.511 (4)	0.442 (3)	0.811 (2)	0.021 (6)*	
H5	0.075 (4)	0.373 (3)	0.533 (2)	0.027 (7)*	
H6	0.266 (3)	0.253 (3)	0.405 (2)	0.020 (6)*	
H8	0.177 (3)	0.419 (3)	1.050 (2)	0.019 (6)*	
H9	0.316 (4)	0.241 (3)	1.174 (3)	0.025 (7)*	
H11	0.201 (3)	−0.113 (3)	0.889 (2)	0.014 (6)*	

### Atomic displacement parameters (Å^2^)

**Table:**

	*U*^11^	*U*^22^	*U*^33^	*U*^12^	*U*^13^	*U*^23^
Br1	0.02086 (9)	0.01838 (10)	0.02144 (10)	0.00506 (7)	0.01007 (7)	0.00289 (7)
Cl1	0.0235 (2)	0.0159 (2)	0.01452 (19)	0.00065 (16)	0.00259 (16)	−0.00138 (15)
Cl2	0.01585 (19)	0.0236 (2)	0.0246 (2)	0.00547 (16)	0.00383 (16)	0.01331 (18)
S1	0.01517 (18)	0.01071 (18)	0.01559 (19)	0.00379 (14)	0.00343 (15)	0.00253 (14)
O1	0.0223 (6)	0.0124 (6)	0.0186 (6)	0.0010 (5)	0.0036 (5)	−0.0010 (5)
O2	0.0210 (6)	0.0179 (7)	0.0223 (7)	0.0101 (5)	0.0054 (5)	0.0071 (5)
O3	0.0125 (5)	0.0132 (6)	0.0159 (6)	0.0027 (4)	0.0023 (4)	0.0044 (5)
C1	0.0160 (7)	0.0112 (7)	0.0168 (8)	0.0032 (6)	0.0072 (6)	0.0034 (6)
C2	0.0134 (7)	0.0167 (8)	0.0192 (8)	0.0030 (6)	0.0024 (6)	0.0054 (7)
C3	0.0146 (7)	0.0158 (8)	0.0150 (8)	0.0010 (6)	0.0002 (6)	0.0024 (6)
C4	0.0139 (7)	0.0104 (7)	0.0151 (8)	0.0026 (6)	0.0035 (6)	0.0021 (6)
C5	0.0136 (7)	0.0158 (8)	0.0151 (8)	0.0022 (6)	0.0002 (6)	0.0024 (6)
C6	0.0167 (8)	0.0167 (8)	0.0138 (8)	0.0010 (6)	0.0013 (6)	0.0029 (6)
C7	0.0123 (7)	0.0120 (7)	0.0146 (7)	0.0025 (6)	0.0043 (6)	0.0035 (6)
C8	0.0185 (8)	0.0141 (8)	0.0145 (8)	0.0021 (6)	0.0031 (6)	0.0008 (6)
C9	0.0166 (8)	0.0188 (9)	0.0148 (8)	0.0004 (7)	0.0017 (6)	0.0028 (7)
C10	0.0110 (7)	0.0163 (8)	0.0191 (8)	0.0032 (6)	0.0038 (6)	0.0088 (6)
C11	0.0136 (7)	0.0124 (8)	0.0192 (8)	0.0033 (6)	0.0053 (6)	0.0040 (6)
C12	0.0123 (7)	0.0137 (8)	0.0128 (7)	0.0010 (6)	0.0035 (6)	0.0005 (6)

### Geometric parameters (Å, °)

**Table:**

Br1—C1	1.8909 (17)	C4—C5	1.392 (2)
Cl1—C12	1.7306 (18)	C5—C6	1.387 (3)
Cl2—C10	1.7361 (18)	C5—H5	0.96 (3)
S1—O1	1.4267 (15)	C6—H6	0.91 (3)
S1—O2	1.4279 (14)	C7—C8	1.386 (2)
S1—O3	1.6163 (13)	C7—C12	1.390 (2)
S1—C4	1.7520 (18)	C8—C9	1.390 (3)
O3—C7	1.407 (2)	C8—H8	0.96 (3)
C1—C2	1.390 (3)	C9—C10	1.387 (3)
C1—C6	1.391 (3)	C9—H9	0.94 (3)
C2—C3	1.391 (3)	C10—C11	1.389 (3)
C2—H2	0.95 (3)	C11—C12	1.387 (2)
C3—C4	1.394 (2)	C11—H11	0.91 (2)
C3—H3	0.94 (3)		
			
O1—S1—O2	120.61 (9)	C5—C6—C1	119.11 (17)
O1—S1—O3	108.71 (8)	C5—C6—H6	120.8 (16)
O2—S1—O3	102.04 (8)	C1—C6—H6	120.0 (16)
O1—S1—C4	109.04 (8)	C8—C7—C12	120.82 (16)
O2—S1—C4	110.81 (9)	C8—C7—O3	120.41 (16)
O3—S1—C4	104.20 (8)	C12—C7—O3	118.60 (16)
C7—O3—S1	119.14 (11)	C7—C8—C9	119.49 (17)
C2—C1—C6	121.93 (16)	C7—C8—H8	119.2 (15)
C2—C1—Br1	118.56 (13)	C9—C8—H8	121.3 (15)
C6—C1—Br1	119.51 (14)	C10—C9—C8	119.06 (17)
C1—C2—C3	119.00 (16)	C10—C9—H9	122.2 (16)
C1—C2—H2	120.7 (16)	C8—C9—H9	118.7 (17)
C3—C2—H2	120.3 (16)	C9—C10—C11	122.05 (17)
C2—C3—C4	119.06 (17)	C9—C10—Cl2	119.28 (15)
C2—C3—H3	123.1 (16)	C11—C10—Cl2	118.66 (14)
C4—C3—H3	117.8 (16)	C12—C11—C10	118.29 (17)
C5—C4—C3	121.71 (16)	C12—C11—H11	121.4 (15)
C5—C4—S1	119.34 (13)	C10—C11—H11	120.2 (15)
C3—C4—S1	118.94 (14)	C11—C12—C7	120.28 (16)
C6—C5—C4	119.18 (16)	C11—C12—Cl1	119.48 (14)
C6—C5—H5	121.9 (17)	C7—C12—Cl1	120.22 (14)
C4—C5—H5	118.9 (16)		
			
O1—S1—O3—C7	−50.31 (14)	C2—C1—C6—C5	−0.6 (3)
O2—S1—O3—C7	−178.76 (13)	Br1—C1—C6—C5	179.19 (14)
C4—S1—O3—C7	65.86 (14)	S1—O3—C7—C8	72.63 (19)
C6—C1—C2—C3	0.8 (3)	S1—O3—C7—C12	−112.06 (16)
Br1—C1—C2—C3	−179.02 (14)	C12—C7—C8—C9	0.9 (3)
C1—C2—C3—C4	−0.5 (3)	O3—C7—C8—C9	176.11 (16)
C2—C3—C4—C5	0.0 (3)	C7—C8—C9—C10	−1.1 (3)
C2—C3—C4—S1	−179.30 (14)	C8—C9—C10—C11	0.6 (3)
O1—S1—C4—C5	−162.54 (14)	C8—C9—C10—Cl2	−178.65 (14)
O2—S1—C4—C5	−27.52 (17)	C9—C10—C11—C12	0.2 (3)
O3—S1—C4—C5	81.53 (15)	Cl2—C10—C11—C12	179.41 (13)
O1—S1—C4—C3	16.82 (17)	C10—C11—C12—C7	−0.4 (3)
O2—S1—C4—C3	151.84 (15)	C10—C11—C12—Cl1	−179.04 (13)
O3—S1—C4—C3	−99.11 (15)	C8—C7—C12—C11	−0.1 (3)
C3—C4—C5—C6	0.1 (3)	O3—C7—C12—C11	−175.43 (15)
S1—C4—C5—C6	179.47 (14)	C8—C7—C12—Cl1	178.50 (14)
C4—C5—C6—C1	0.2 (3)	O3—C7—C12—Cl1	3.2 (2)

### Hydrogen-bond geometry (Å, °)

**Table:**

*D*—H···*A*	*D*—H	H···*A*	*D*···*A*	*D*—H···*A*
C3—H3···O1	0.94 (3)	2.51 (3)	2.919 (2)	106.2 (18)
C2—H2···O3^i^	0.95 (3)	2.58 (3)	3.482 (2)	160 (2)
C11—H11···O1^ii^	0.91 (2)	2.52 (2)	3.390 (2)	160 (2)

Symmetry codes: (i) *x*+1, *y*, *z*; (ii) *x*, *y*−1, *z*.
